# Co-constructing effective collective intelligence networks in rare diseases: a mixed method approach to identify the parameters that matter for patients, professionals and policy-makers, piloted in Cyprus

**DOI:** 10.1186/s13023-023-02672-y

**Published:** 2023-04-28

**Authors:** Victoria Antoniadou, Adamos Hadjipanayis

**Affiliations:** 1 Cyprus Alliance for Rare Disorders, Akropoleos 71, Nicosia, 2012 Cyprus; 2 Medical School, European University Cyprus, Karyatidon 42, Larnaca, 6042 Cyprus

**Keywords:** Rare Diseases, eHealth, Healthcare Networks, Patient networks, Patient empowerment, Continuing Medical Education

## Abstract

**Background:**

Rare diseases are a particular field of public health that is characterized by scattered, often insufficient knowledge and infrastructure. The scarcity of specialized knowledge often forces clinicians and patients to an incomplete picture of the diseases and their associated risks. Effective person-centred networks appear promising for solving such real world and life-defining problems by purposely sourcing expert knowledge that is geographically-dispersed. The design and implementation of the RARE-e-CONNECT network technology is described. The project was funded to create collaborative spaces for the development of international partnerships in Cyprus’ healthcare, promoting the dissemination of expert knowledge on rare diseases while saving resources through teleconsultation. Parameters that matter for patients, providers and policy-makers through the RARE-e-CONNECT experience were evaluated through a participatory mixed-method approach, consisting of (1) a needs assessment survey with 27 patients/families and 26 healthcare professionals at the two referral hospitals for the diagnosis and management of rare diseases in Cyprus; (2) interviews with 40 patients, families and patient representatives, as well as 37 clinicians and laboratory scientists, including national ERN coordinators/members; (3) activity metrics from 210 healthcare professionals and 251 patients/families/patient representatives who participated on the platform at the time of the research.

**Results:**

Our results indicate usage and intention by both healthcare professionals and patients/families to openly provide decentralized specialized information for raising suspicion amongst clinicians to facilitate the necessary referrals, as well as peer to peer psychosocial support to help cope with the everyday challenges of living with the disease. User behavior was largely affected by the prevailing social norm favoring individual practice, as well as missing policies for telemedicine and shared care. This article discusses how telehealth is inextricably linked to social, cultural, organizational, technological and policy factors affecting uptake.

**Conclusions:**

We argue that collective intelligence tools need to be formally considered and work hand in hand with national and European policies/regulatory frameworks to promote proactiveness amongst the healthcare community with regard to the timely diagnosis of rare diseases and the facilitation of patients’ pathway to specialists. Collaborative channels between countries need to be established to source collective intelligence on complex cases and save resources through teleconsultation/telementoring.

## Background

Collective intelligence (CI) emerges when many individuals work together online, offline or in blended formats, exchanging knowledge and experience to solve problems of mutual concern. The idea is to create BIG MIND situations based on cognitive diversity, independence, utilization of decentralized knowledge, and effective aggregation of dispersed knowledge [31] [24] to help find solutions to manage life-threatening conditions and solve public health problems in the long run.

In rare diseases, effective collaborative networks, as one instance of telehealth^1^, play a key role in addressing the shortage of expertise by advancing Continuing Medical Education (CME) in line with the latest developments, research and evaluation [36]. Collaborative networks are able to share valid research knowledge and clinical expertise in the form of teleconsultation/telementoring between expert and less expert physicians across geographies [37]; thus seem to be very much in line with public health goals to tackle the challenges of timely diagnosis and effective management of rare diseases according to the available curative solutions [16] [28] [22].

Patients learn at the doctor’s office, conferences, online and empirically, while doctors learn through conferences, published research, case discussions and empirically. Combined, the CI and Medicine 2.0 principles, featuring increased openness of (specialized) information, collaboration and apomediation, appear promising for creating extended learning situations [21] [12] [29].

Nonetheless, authors such as Mulgan [20] caution about how far we are from achieving a truly global CI able to solve global problems such as rare diseases, despite the enormous potential that derives from the existing Internet and social media practices. The author points out the need for concerted action to “assemble new combinations of tools” to support world thinking and acting in ways and paces analogous to the existing problems, contextual relevancies, policies and structures that will inevitably determine their meaning(fulness) and applicability.

Clearly, there is no universal guide or tool for creating successful technology-supported information networks to bring together a critical mass of geographically scattered expertise and promote interaction in such a way as to effectively drive CI. The degree to which meaningful content is provided, the extent to which a technology is able to respond to the needs and contextual relevancies, and the level of visibility it receives are key determinants of a network’s adoption and success [30].

This article reports on findings from the empirical application of the RARE-e-CONNECT network technology platform. This network technology sought to create electronic collaborative spaces to support national and international partnerships in Cyprus’ rare disease patient care. The RARE-e-CONNECT project was co-financed by the European Regional Development Fund and the Republic of Cyprus through the Research and Innovation Foundation under the grant name POST-DOC/0916/0222, for a duration of 3 years (2019–2022). It involves the creation of (online) networks of patients exchanging information about similar problems, as well as networks of clinicians and other healthcare professionals exchanging information and collaborating on complex cases [19].

The networks are technologically implemented as 25 patient communities and 25 healthcare communities organised by disease group e.g., neurological conditions. There is an additional community for general discussions, one for patients/families and one for professionals. This community is conceived as a non-disease specific CI forum for the discussion of disease-agnostic aspects e.g., diagnostic challenges, visible or invisible disabilities, social exclusion. Each forum is accompanied by an individual mailing list to each audience. Other functionalities for healthcare professionals include medical teams through which they can collaborate on topics of their choice or situations they have at hand (e.g., complex case management). They can select colleagues on the platform based on profile information or invite colleagues based on previous collaborations or other criteria. For patients, there is an interactive wall hosting patient stories. For patients and healthcare professionals alike, there is a specialist centre repository to facilitate cross-border healthcare, a webinar repository, and a doctor-patient forum. All functionalities are accessible via a single online space, which is the RARE-e-CONNECT platform.

The RARE-e-CONNECT model is conceived as an innovative model of telehealth for patient care and education, extending opportunities for medical and patient education through peer mentoring in real time and asynchronously. The implementation of this project coincided with the creation of the European Reference Networks (ERNs), and ran in parallel with national needs for participation in the ERNs. Thus, the goals of this project included the dissemination of specialised information produced by the ERNs into the local healthcare and patient community [11]. Specialised information was, for example, guidelines on several rare diseases, specialist centres by disease group and diseases and the like. Locally, the implementation of this project coincided with the two-phased implementation of the Cyprus National Health System in 2019 and 2020.

This project had a strong research component to investigate the impact of telecollaboration (virtual exchange) in terms of enhancing the goals of patient-oriented rare disease healthcare. The results of this knowledge-sourcing activity would be analysed and channeled to decision-makers to assist in the formulation of national RD-related standards, which do not currently exist.

We were interested in determining important parameters not only of the technological design but also its adoption by the community. This process was guided by the principles of the enhanced exploratory framework proposed by Chen et al. (2020), bringing together Community-Based Participatory Research and Human-Centred Design (CBPR/HCD) [4].

## Methods

To achieve our research objectives, not a purely numerical approach to data collection and analysis was used. Instead, multiple research tools were used, consisting of surveys, focal interviews, activity metrics and field notes, to capture rich data from patients, families, healthcare professionals and national authorities at various stages and timeframes that questionnaires alone would not be able to capture.

This approach allowed researchers to delve into the real work and life settings of the rare disease provider and patient communities, record their needs, habits and experience and involve them in the construction of this network [1]. In turn, it allowed us to conduct a bottom-up mixed method analysis with a variety of data and perspectives, and make rich theorizations about the parameters that matter to our target groups, at all stages underlying the development of the RARE-e-CONNECT network technology [30].

### Needs assessment survey

A needs assessment survey was carried out in 2019, soon after the launch of the project. We contacted (1) individual patients/families and the rare disease patient associations registered at the Cyprus Alliance for Rare Disorders (CARD) and (2) healthcare professionals at the two largest hospitals in Cyprus, paediatric and adult, who were directly involved in rare disease diagnosis and management. Our objective was to confirm the relevance of the original proposal with groups of individuals that were considered the “extreme users” of the platform-to-be, or adjust our plans accordingly [4].

Both quantitative and qualitative data were collected by means of questionnaires consisting of 13 closed- and 4 open-ended questions about current collaborative practices, Web 2.0 literacy, diagnostic and management needs, and CME. Questionnaires were completed by 26 healthcare professionals and 27 patients/families/patient representatives during in-situ presentations of the project, leading discussions and providing further qualitative information on the networking/data sharing needs and expectations from the RARE-e-CONNECT technology-to-be.

### Focal interviews

Interviews were conducted one-to-one with:

- thirty-seven key healthcare professionals, including primary care physicians and rare disease specialists. Some of the specialist clinicians and laboratory scientists interviewed were also national leads or affiliated with the ERNs^2^ in which Cyprus participates, as well as allied healthcare professionals^3^.
- forty patients and family members, including patient representatives collaborating with the Cyprus Alliance for Rare Disorders.
- competent bodies in Cyprus, i.e., the Ministry of Health (MOH), the National Representative at the ERN Board of Member States and the Cyprus Medical Association (CyMA).

Where permission was granted, the interviews were audio-recorded. Otherwise, we kept extensive field notes that included quotes and word by word text. The interview data were real world data, consisting of information, not just about technology usage but also about the wider framework underlying the initiative, policies and other specifics. As such, they were duly transcribed and systematically analysed in three stages or levels of coding [5], as per the grounded theory approach:

1. Open coding (conceptual labelling, discerning quotes and meaning chunks).
2. Axial coding (finding relationships between open codes and categorising).
3. Selective codes (relating to theoretical constructs).

In our case, we drew on the theoretical constructs of the Unified Theory of Acceptance and Use of Technology (UTAUT) [30], Medicine 2.0 [12] [29], and CI [20] to interpret the most frequent (core) categories that derived from our data.

### Objective data of actual use (activity metrics)

We gathered quantitative activity data from 251 patients/families and 210 healthcare professionals from 23.09.2021 to 23.09.2022. This data illustrated the actual usage/user behavior around the functionalities proposed, i.e., Registration/Profile, Patient Communities, Healthcare Communities, Medical Teams, and Patient Stories.

Overall, the aim of the analysis was not to try to prove or disprove a predefined hypothesis about the benefit of this technological proposal [7] but to develop an objective and contextualized theory of how this proposal could be useful for promoting progress in rare diseases in relation to the latest advances in the European arena, i.e., the Integration of European Reference Networks into National Healthcare Systems; European Health Data Space (EU4HealthWorkProgramme, 2022, 61:62^4^ [26]. To eliminate any bias, we triangulated our codes and findings across multiple data sources collected at different timeframes [10] [18] [27].

## Results

### Needs assessment survey

**Fig. 1:**
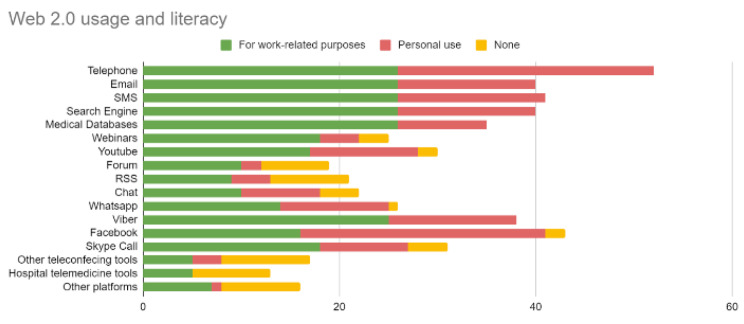
Healthcare professionals’ collaborative practices

**Fig. 2:**
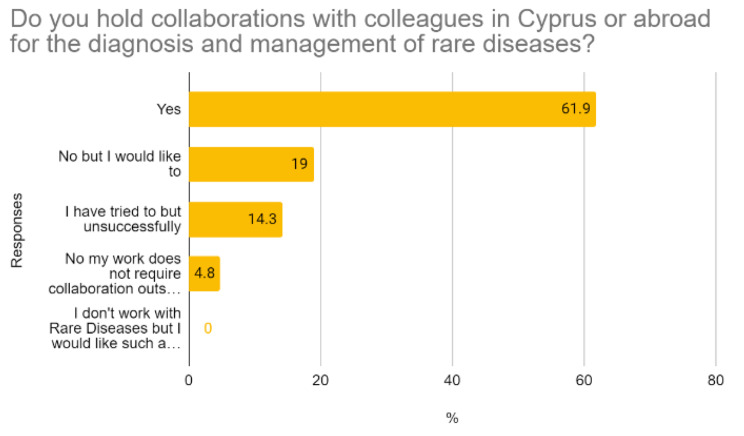
Healthcare professionals’ usage of Web 2.0 implying literacy

Figures 1 and 2 depict the percentage of the surveyed healthcare professionals holding collaborations in 2019, as well as their Web 2.0 tool usage for professional and other purposes. This data revealed remaining needs despite existing collaborations, willingness to expand collaborations, and digital literacy.

**Fig. 3:**
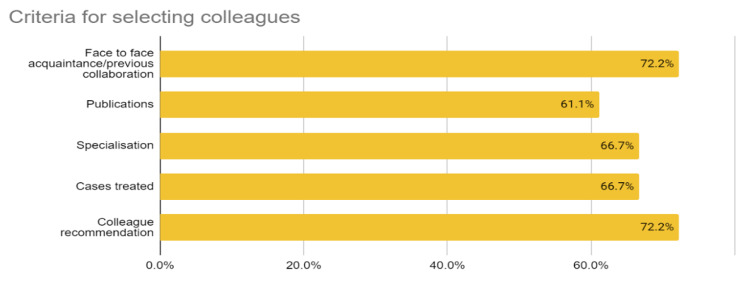
Healthcare Professionals’ criteria for selecting colleagues

This research also sought to know the professionals’ main criteria for choosing their colleagues. As expected, their selection criteria were face-to face acquaintance, degree of specialisation, publications, previous collaboration or recommendation by other colleagues (Fig. 3).

**Fig. 4:**
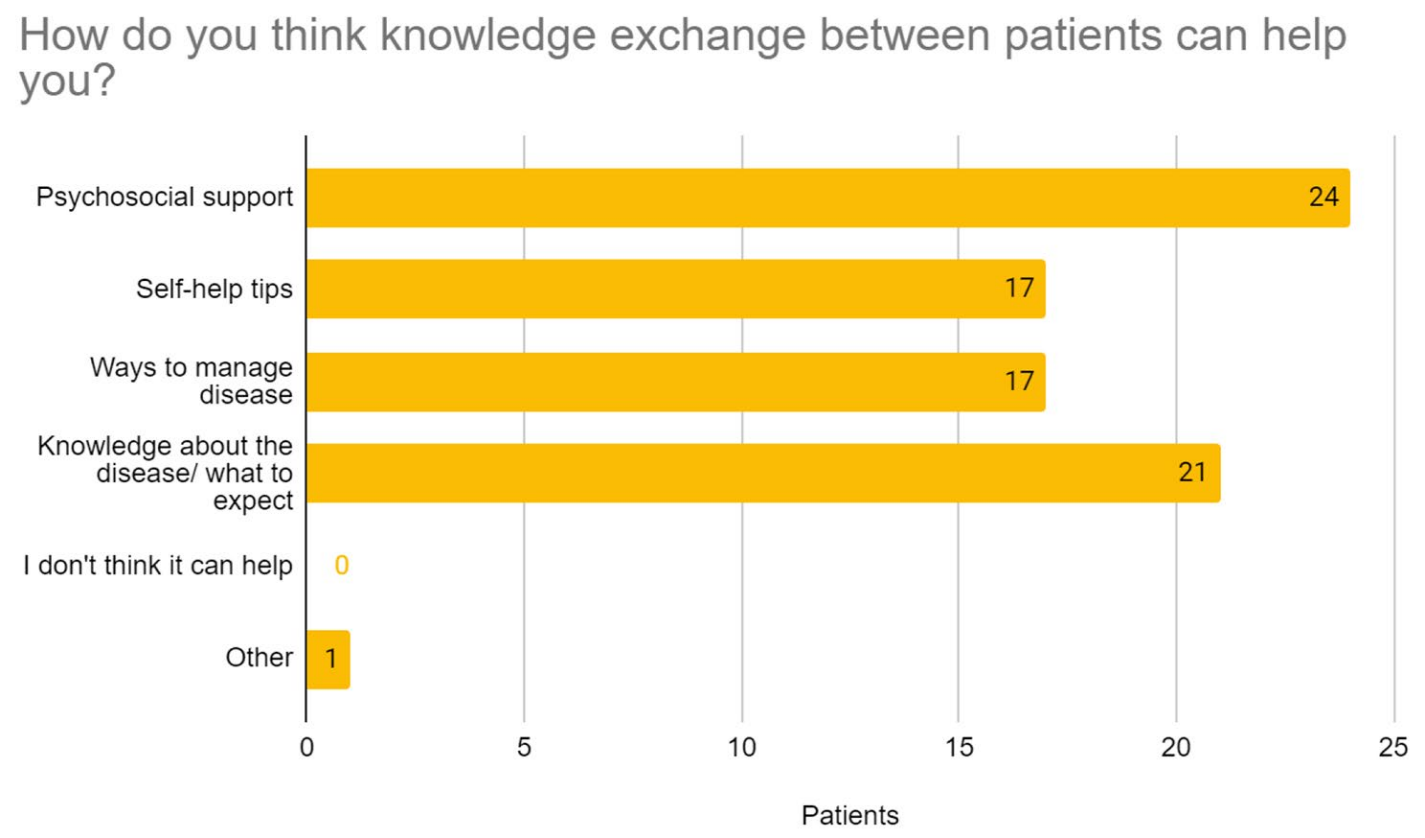
Patients’ and families’ perception of help received through patient networking

**Fig. 5:**
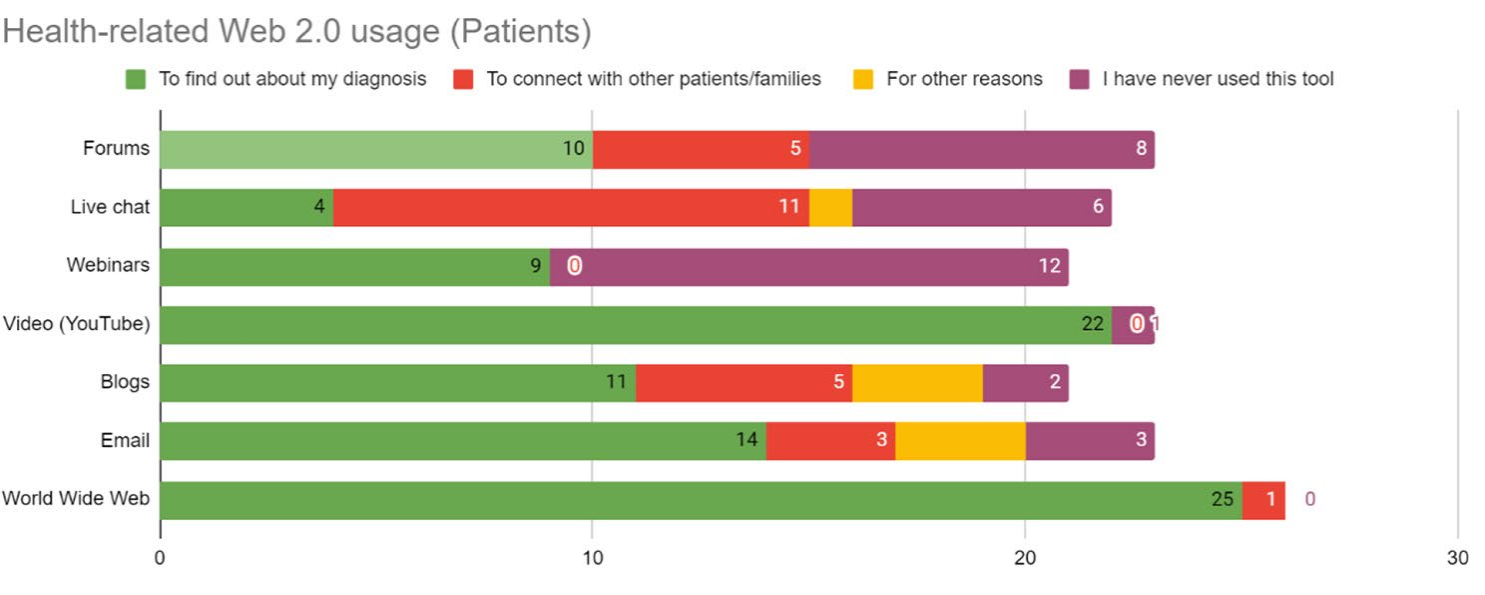
Health-related Web 2.0 usage of patients/families

From the patients/families’ side, it was confirmed that sharing knowledge and experience with other patients/families could provide them with psychosocial support and better knowledge about the disease itself, including self-help tips (Fig. 4). They largely used the Internet and Internet applications to find health-related information, as elaborated in Figs. 4 and 5.

### Qualitative data analysis (interviews)

The real bulk of information in terms of the parameters that matter for healthcare professionals (general and specialists), patients/families and national authorities was achieved through the interviews that were carried out post-launch of the RARE-e-CONNECT network technology and its pilot in the real life and work settings of our target groups.

Overall, the analysis of our qualitative data generated a total of 1357 codes (pieces of coded text), which were categorized under 237 quirks (titles) using the Quirkos software. Each data source generated a different number of codes (see Table 1).

**Table 1:** Number of quotes (coded text) per source

No.	Source	Quotes #	Objective/ Stage
1	HCPs_Needs analysis (open-ended questions)	26	Needs assessment
2	Patients_Needs analysis (open-ended questions)	211	Needs assessment
3	Meeting Minutes MOH-CARD	7	Needs assessment
4	CYMA Minutes - FINAL	14	Evaluation of intention
5	Patient interviews	279	Evaluation of intention
6	HCP interviews	408	Evaluation of intention
7	Posts on RARE-e-CONNECT (Patient stories)	363	Usage
8	Doctor-patient forum posts	13	Usage
9	HCPs forum posts	8	Usage
10	Patient forum posts	28	Usage
**TOTAL**		**1357**	

The 1357 open codes were categorized under the 5 categories of actual use, performance expectancy, effort expectancy, social influence and enabling conditions, proposed by Venkatesh et al. (2003) [30] in the UTAUT framework. As per the grounded theory principles, these categories were used to develop a storyline discussion of the findings. Concepts from Medicine 2.0 and the CI framework were also used where appropriate to interpret our findings [3] [13] [14] [20] [22] [24] [29]. Due to space constraints, here we present the more frequent open codes under each category.

With regard to the actual use of the system by healthcare professionals and patients/families, our qualitative findings are summarized in the subcategories listed in Table 2.

**Table 2:** Selection of codes indicating actual system usage by patients/families and healthcare professionals by frequency

1. **Describing journey to diagnosis (frequency: 136/1357)**
Late diagnosed; Nobody believed me; Sharing first signs and symptoms; doctors’ key role in breaking bad news; Distressing journey through differential diagnoses and process of elimination; Misdiagnosed, mistreated or not diagnosed locally
2. **Being a mentor to others (frequency 147/1357 codes)**
Need disease knowledge and psychological support that only a patient can offer; Sharing information about treatment; Don’t ever think you are alone - Parents and patient fighters and winners; Coping mechanisms with the disease
3. **Everyday challenges (frequency 145/1357 codes)**
Talking about the discrimination they experience because of their disease; Diagnosis putting life on hold– work, school, studies, going abroad, leisure; Difficult to explain a disease that people cannot see; Sharing emergency experiences; Finding ways to communicate with non-verbal children; Helping rd children develop autonomy - long term
4. **Specialists share expertise (frequency 12/1357 codes)**
L-carnitine dosage to avoid unpleasant side effects; Carglumic acid for chronic hyperammonemia; Gene therapy options for IMD; Gene therapy for neuromuscular diseases; Why does the number IMDs increase over time?; What patients need to know about Primary Ciliary Dyskinesia; New therapies for Tourette; Genetics vs. Clinical criteria of FMF; Emergency guidelines for rare diseases – Orphanet; Preimplantation Genetic Diagnosis; PIDs early detection - signs and symptoms

Each of the open codes under subcategory 1, titled “Describing journey to diagnosis”, contains instances where patients/families related an aspect of their journey to diagnosis. The codes under subcategory 2, titled “Being a mentor to others”, contain instances where patients/families act as mentors to others, relating lessons learned from experience with the condition. Each of the codes under subcategory 3, titled “Everyday challenges”, contains instances where patients/families related everyday challenges of living with the disease, providing awareness and self-help tips for others found in similar situations. Each of the codes under subcategory 4, titled “Specialists share expertise”, contains instances where specialists answered patients’ disease-specific questions in the doctor-patient forum e.g., available treatments. Also under this subcategory are instances where clinicians and laboratory scientists used the professional forums to share disease-related information or, locate colleagues for multidisciplinary collaboration, e.g., psychologists.

Healthcare professionals’ usage of the system also had to do with the first signs of a rare disease, e.g., Genetics vs Clinical criteria of Familial Mediterranean Fever (FMF). Laboratory scientists contributed knowledge on diagnostic and curative methods, currently available or under development, i.e., preimplantation genetic diagnosis, innovative research under development, gene therapy and access.

These qualitative findings showcase how the RARE-e-CONNECT network technology became a place for patients/families to describe the first signs and symptoms of the disease, as they experienced them. Many patients stressed how their “case wasn’t a textbook case”, happening earlier than described in the literature, causing doubts to their treating physicians. Others stressed how doctors wrongly attributed signs and symptoms to prematurity of birth and other factors. Very importantly, patients and parents used the platform to talk about their experience with new methods of genetic diagnosis, share concepts and experiences related to genetic conditions, talk about how they sought professional psychological support, etc. (subcategory 2). Such information may help break taboos typically related to a rare (genetic) disease diagnosis. In subcategory 3, the semantic link between the codes is the everyday challenges deriving from the condition. Patients/families talk about how the diagnosis puts life, i.e., work, school, studies, leisure on hold, and creates multiple needs for coping mechanisms, e.g., finding ways to communicate with non-verbal children and help them to become more independent and develop life skills for when the parents will no longer be present.

Overall, the findings in these 4 subcategories can be taken as an example of how patients and specialists can, through sharing their experience with the condition and expertise, create bulks of decentralized knowledge related to rare disease diagnosis to serve CI on rare diseases, lead research and expansion.

### Performance expectancy

Performance expectancy is defined by Venkatesh et al. (2003) [30] as the degree to which the group believes that using the system will help them attain gains in their job performance. There were 368 out of the 1357 open codes derived from our qualitative analysis, referring to performance expectancy by healthcare professionals and patients/families. A selection of these open codes is provided in Table 3. Each of these codes contains instances where healthcare professionals and patients indicated a way in which the platform could be compatible with their job and needs.

**Table 3:** Selection of open codes referring to performance expectancy, based on frequency

**Performance expectancy of tool for healthcare professionals and patients/ families (frequency 368/1357 codes)**
Raise suspicion - Increase GPs and specialists’ awareness/knowledge on rare diseases: “Myth busting”; Comprehensive, easy to use and adaptable tool for networking and multidisciplinary collaborations; Information/discussions about ultra-rare diseases complex cases involving multiple autoimmune; The real added value would be Europeans coming in otherwise too small of a number for expertise to grow and patients to network; Original content/interaction you cannot find on websites; HCP learning from patient experience because the disease doesn’t manifest in the same way for all patients; Large scale dissemination of specialized information; Needing to learn about new therapies/ clinical trials and how I can participate; Opportunity for patients to come out: Is my diagnosis rare?; Needing access to specialist repository by disease group and disease; Opportunity for patients to come out- work in progress

These instances could be taken to relate directly with findings from the usage of the platform, as described above, particularly the instances where both groups perceived that using this network technology could help “Raise suspicion amongst frontline healthcare professionals to facilitate timely diagnosis and lead effective management” according to the available curative options. Specialists were particularly fervent about the need for “myth busting” regarding rare diseases and “being proactive for timely diagnosis, and not reactive”, identifying this latter as “the problem of the medical community”.

Also, healthcare professionals and patients clearly called for original content/specialized information about unknown ultra-rare diseases, “going beyond dry information easily available on websites”. For the healthcare professionals in particular, “the real added value would be Europeans coming in who are hard to find on the phone” to discuss complex cases where there is no expertise locally and for which the limited number of cases in small EU countries, such as Cyprus, does not favor substantial development in terms of the required expertise.

The possibility of having large-scale dissemination of specialized information through this network technology platform was particularly valued, by both patients/families and provider groups, for materializing the need to raise clinical suspicion about rare diseases. This allowed us to draw conclusions on the objective usability [30] of this system in relation to other systems used by physicians and patients/families.

From their perspective, patients/families deemed particularly important to have disease-related information for ultra-rare diseases in “layman terms” and “native language” to cope with the difficulty involved in understanding and processing complex medical terms from various sources and “not only from their doctor”. Professionals also acknowledged a significant gap in this area, seeing the network as an “opportunity to “lead informed decision-making”, with relevant and trustworthy health information beyond the hospital space (“apomediation”) [13]. For patients, perceived usefulness indicating performance expectancy, was also inextricably related to the ability to “share with people who know what they are talking about” and were able to give them “self-help tips” for managing the everyday of the disease, as well as practical information for traveling abroad for treatment, for example. As expected, a major patient need was to ask other patients about specialists who had helped them with the diagnosis and/or management of the condition, in Cyprus and/or abroad.

Healthcare professionals’ demand for a specialist finder was twofold: (1)  to help them locate colleagues in Cyprus by specialty area and services as well and (2) this colleague finder to also be visible to patients/families (this was not entirely possible at the time of the research, in the absence of national accreditations and with limited to no participation of national centres in the ERNs).

### Effort expectancy

Effort expectancy is defined by Venkatesh et al. (2003) [30] as the degree of ease associated with the use of the system. There were 75 out of the 1357 open codes derived from our qualitative analysis referring to effort expectancy. A selection of open codes in this category is provided in Table 4. Each of these codes contains instances where healthcare professionals and patients indicated their perception of the effort required for using the network technology.

**Table 4:** Selection of open codes referring to effort expectancy, based on frequency

**Effort expectancy (frequency 75/1357 codes)**
Notifications to promote visibility of content; Ι see patients all day long at the office, I wouldn’t want to keep receiving messages at night; People don’t have time to write long texts; Need dedicated time for this within work schedule but I will do it; Video audio image modality; We need someone to organise us to do this; Interoperability with other platforms

This meant that, despite the perceived ease of use of the technological configuration, the system was found to be largely incompatible with the time demands of the workplace and personal life, demanding effort to decide on content and present complex information in writing. Time constraints were also quoted as the cause of no participation in other collaborative initiatives outside the RARE-e-CONNECT project. At the same time, the largely written modality of the RARE-e-CONNECT network technology was found to add to the amount of effort that the participants perceived they had to put into participating.

Both groups, patients/ families and healthcare professionals, identified the need to have “strong patient support groups to properly share specialised knowledge”, indicating shortcomings in trained staff, digital infrastructure and literacy. Nevertheless, a significant number of specialists and patients/families formulated plans to share specific content related to their specialization or diagnosis later on. Venkatesh et al. (2003) [30] call this “behavioral intention” and define it as “the degree to which a person has formulated conscious plans to perform or not to perform some specified future “behavior”. Behavioral intention is aligned with factors such as the subjective norm (social influence), image, job relevance, output quality and result demonstrability.

Administrative support/community managers were clearly needed to identify meaningful content, organising and animating writing.

### Social influence

Social influence is defined as the extent to which individuals perceive that important others feel that they should use the new/target system [30] and it is a very influential factor in the adoption of a new system. Derived from our qualitative analysis, there were 129 open codes out of the 1357 open codes referring to aspects of social influence affecting uptake and participation. A selection is provided in Table 5.

**Table 5:** Selection of open codes referring to social influence, based on frequency

**Social influence (frequency 129/1357 codes)**
Directing to colleagues indicating hierarchies and status-quo; Too small of a number for a health professional to focus on/ develop expertise; Perceived interest or lack of interest from important others; Fiefdoms - Individual thinking/practice; Immediacy of information and momentum; Some patients don’t want to share about disease - Taboo

Each of these codes contains instances with a semantic link to aspects of social influence i.e., mentality constructs, perceived interest or lack of interest from important others, taboo issues, incentivizing them to register and actively participate on the platform or not.

For healthcare professionals, important others were their colleagues in Cyprus and colleagues from Europe, while important others for patients/families were other patients/families with the same diagnosis.

A particularly inhibiting factor for healthcare professionals and patients/families was local mentality. According to the healthcare professionals and patients/families who participated in our research, local mentality was characterised by “Fiefdoms - Individual thinking/practice”, “hierarchies and existing status-quo”, “Doctors know it all” as well as “doctors need to know it all” mentalities by doctors and patients respectively, and the stereotypical patient perception that “Foreign doctors are never wrong”.

The doctor-patient forum, for example, was created to provide opportunities for multiple physicians to respond to a patient question and vice versa, helping CI to grow. However, it was mentioned that the fact that other healthcare professionals could see a professional’s response to a patient could potentially cause misunderstandings between colleagues. As a result, some healthcare professionals called for private chatting to avoid such possibility.

The presence of taboos was also included in this category, which patients and patient representatives acknowledged as an impeding factor that inhibited many patients/families from approaching associations and/or openly talking about their diagnosis. This finding was also evident in the ways the participants used the option of anonymity on the platform (see Table 6).

**Table 6:** User behavior from 23.09.2021 to 23.09.2022

Activity	Patients/ Families	Healthcare professionals
Registrations	251	210
Anonymity	Fully Anonymous 50 Semi Anonymous 139 Not Anonymous 62	N/A
Profile completion	126	106
Joined communities	240	151
Number of posts	84	24
Number of resources	9	1
Number of replies to posts	122	5
Number of patient stories	25	N/A
Number of replies to patient stories	7	N/A
Medical Teams (creation + colleagues)	N/A	23
Medical teams (request meeting)	N/A	0

### Enabling conditions

Venkatesh et al. (2003) [30] define enabling conditions as the degree to which an individual believes that an organizational and technical infrastructure exists to support use of the system and the determinants of any user behavior.

In this research, 344 out of the 1357 open codes derived from our qualitative analysis were semantically linked to enabling or inhibiting conditions for the adoption of the RARE-e-CONNECT network technology. A selection of the most frequent open codes under this category is provided in Table 7.

**Table 7:** Selective coding and frequency for enabling conditions

Enabling (or inhibiting) conditions (344/1357 codes)
1. **Experience (frequency 84/1357 codes)**
Previous experience connecting with other patients; Not only from my doctor: I communicate with specialists abroad for my child’s needs; Experience with national networking platforms abroad; Lived experience with rare diseases/ERN collaboration abroad; Access but no real collaboration with ERNs up to now; Each patient case is different – Misinformation; Used to phone and email for my communications but this is not multidisciplinary collaboration; Experience with national networking platforms abroad (configuration of tools); Reading vs. contributing culture
2. **Lack of “hedonic pleasure”/disease-related conditions (frequency 44/1357 codes)**
Psychological burden from negative aspects of disease; “We don’t want to talk about the disease all the time”; “If I had something I would know by now”; People usually come when they need something/ they are facing an emergency with regards to treatment / financial support
3. **Indicating external to the technology variables determining their user behavior (Policies) (frequency 94/1357 codes)**
No departments/centres of expertise and patient pathways; Need for nationally-accredited diagnostic labs; Need telemedicine for follow ups in collaboration; Reimbursement of shared care; Unsure about legal aspects for sharing scientific information from networks
4. **Indicating inherent and external to the technology variables determining their user behavior (infrastructure) (frequency 95/1357 codes)**
Difficult to create multidisciplinary teams because there is not enough expertise; Need basic supportive hospital structures to be able to participate: Not enough specialised staff for multidisciplinary teams leads clinician’s extended role; Need strong patient support groups

Enabling conditions had to do with previous experience with networks, the presence of a policy framework, organizational and technical infrastructure to support the use of the system, the perception of enjoyment when using the system (which Venkatesh et al. 2003 call “hedonic pleasure”) [30]. As Christensen and Mackinnon (2006) also argue, user characteristics and preferences are important determinants of use and uptake [6].

Each of the subcategories listed in Table 7 contains the most frequent open codes of instances where healthcare professionals and patients/families indicated how they felt enabled or inhibited from engaging in this network technology.

Certainly, the degree to which the healthcare professionals and the patients/families had previous experience using similar tools or were engaged in professional collaborative activities (i.e. ERNs, national networks abroad) affected the degree to which they shared content or made conscious plans to do so, including transferring ERN-related information into the local community, “provided that all policy and legal requirements were met”.

Also related to previous experiences affecting behavior towards the RARE-e-CONNECT network technology and overall project was the finding of healthcare professionals who were hesitant to connect patients to other patients. Considering that “each patient case was different”, they wanted to protect their patients from seeing cases of “frightening” deterioration of the disease, which might not happen to them. This was also evident in the case of patients who had previously participated in patient support groups and found that the disease manifestations were not the same in all patients, advising to be cautious.

Also included in the category of enabling conditions were codes that referred to the emotional burden involved in sharing disease-related information. These codes came exclusively from patients/families who stated that they “didn’t want to talk about the disease all the time”. Interestingly, there was a comparison between RARE-e-CONNECT, Facebook and other social media platforms offering exposure to all kinds of information, disease-related and disease-free, which can also offer enjoyment. This finding suggested that the RARE-e-CONNECT network technology may not be used nonstop but, given its restricted scope to rare disease information, would serve patients in bottleneck or emergency situations (“People usually come in when they need something/ they are facing an emergency with regards to treatment/financial support”). It also suggested that it could be used as part of a configuration of tools, e.g., phone and email, to facilitate patient and provider pathways to information and collaboration, a channel that would increase its meaningfulness and potential as content piled up.

The presence of a compatible policy and organisational framework supporting the use of the system was also coded in our qualitative data. Specifically, healthcare professionals who participated in our research stated that missing or insufficient expertise locally hindered networking possibilities and multidisciplinary collaborations, while “Not enough specialized staff to support clinical work”, i.e., nurses, genetic counselors, led to clinicians having to assume an extended role, leaving them with no time for other activities.

Policy-wise, formal accreditation of national centres of reference, as well as regulations on telemedicine and shared care, were quoted on multiple occasions by healthcare professionals and patients/families alike, as key to defining content reliability and attracting user activity on the RARE-e-CONNECT platform. Specifically, healthcare professionals and patients/families/patient representatives identified the need for meaningful policy making to establish:

(1) departments or centres of reference/centres of expertise and nationally-accredited diagnostic labs, equipped to timely and effectively handle rare disease emergencies, regulate patient referral procedures to specialists locally and, when needed, to European centres of expertise via the national coordinators or otherwise.

(2) telemedicine and shared care regulations, including clear systems evaluating expertise and quality of service, legal vesting for adopting therapeutic protocols and disseminating specialized knowledge from European networks, as well as clear mechanisms for shared care and reimbursement.

For patients/families and healthcare professionals alike, telehealth was clearly needed for following up on patients diagnosed and initially managed abroad. It was also needed for “establishing ways and infrastructures to ensure the development of national expertise to accommodate the needs of the entire patient population”, saving resources and ensuring a viable health system in the long run.

### Activity metrics

Patients and healthcare professionals with the disease profile and specializations listed below were registered on the RARE-e-CONNECT platform at the time of this research (Table 8).

**Table 8:** Participants’ profile

Patient profile	Healthcare professionals’ profile
Oral facial digital syndrome type 6 and Joubert Syndrome; Familial Amyloid Polyneuropathy; Myasthenia Gravis; Ocular Motor Apraxia; PSP - PROGRESSIVE SUPRANUCLEAR PALSY; FSHD Muscular dystrophy; Hereditary neuropathy with pressure palsies (HNPP); Charcot Marie Tooth disease; Friedreich’s ataxia; Νeurofibromatosis Type 1; Multiple sclerosis; Centronuclear myopathy related to dynamin 2 mutations (CNM DNM2); Meige syndrome; Emery Dreifuss Muscular Dystrophy; Generalised Dystonia; Carnitine palmitoyltransferase II deficiency II Myopathic Form (CPT II Myopathic Form); Duchenne Muscular Dystrophy; Limb girdle muscular dystrophy; Spinal Muscular Atrophy (Type I); Parry-Romberg syndrome; Common Variable Immunodeficiency (CVID); CVID with BILU Syndrome; Granulomatosis with polyangiitis (GPA/ Wegener); Familial Mediterranean Fever (FMF); Periodic Fever, Aphthous Stomatitis, Pharyngitis, Cervical Adenitis syndrome (PFAPA syndrome); Still disease; Behçet Disease; Psoriatic Arthritis; Relapsing Polychondritis and Ankylosing spondylitis; Cold agglutinin disease; Lichen planopilaris; Oesofageal achalasia; Median arcuate ligament syndrome (MALS).; Glutaric Aciduria Type 1; Maple serum urine disease; Ornithine transcarbamylase (OTC) deficiency; Phenylketonuria (PKU); GABA transaminase deficiency; Non-ketotic Hyperglycinemia; Darier disease; Cutaneous Mastocytosis (child); Xeroderma Pigmentosum; Familial Adenomatous Polyposis; Von Hippel-Lindau disease; Carney-Stratakis Syndrome (Carney Triad); Medullary thyroid cancer MEN2A associated; Ocular Motor Apraxia; Complete achromatopsia 3; BEST disease; Primary Ciliary Dyskinesia or Kartagener syndrome; Cystic Fibrosis; Lymphangioleiomyomatosis; ADNP syndrome; Kleefstra syndrome; Loeys Dietz Type II; 48XXYY; Congenital Muscle-Brain-Eye Disease; Maternally-Inherited Diabetes and Deafness (MIDD); MIRAGE syndrome; Amyloidosis (secondary); Laryngeal cleft; Tuberous Sclerosis; Williams syndrome; Prader-Willi Syndrome; Adrenal insufficiency (Addison disease); Medullary thyroid cancer MEN2A associated; Biliary atresia; Alpha-1 antitrypsin deficiency; β-thalassaemia; Erdheim-Chester syndrome; Myelofibrosis; Cold agglutinin disease; Erythromelalgia; Thrombophilia; Raynaud disease; Ehlers-Danlos Syndrome; IC (Interstitial Cystitis)	Clinicians: Anaesthesiology; Cardiology; Medical Oncology; Dermatology; General Medicine; Haematology; Clinical Genetics; General Paediatrics; Specialised Paediatrics; Neurology; Nephrology; ENT; Ophthalmology; Nuclear Medicine; Pathology-Oncology, Pulmonology; Radiology-Oncology Laboratory scientists: Molecular Genetics; Biochemical Genetics; Biology Allied health professionals: Genetic counselors; Clinical Psychology; Physiotherapy; Speech Therapy; Occupational Therapy; Clinical Nutrition; Social Work

Table 6 indicates the ways patients/families and healthcare professionals interacted with each feature of the platform.Quantitatively, the patients’ usage of the platform was significantly greater than the usage of the healthcare professionals. Anonymity was important for patients, the majority of whom chose full or semi-anonymity. Our criteria for anonymity were as follows: Fully anonymous: The participant has used a username and full name that do not allow identification by either the rest of the patient members of the platform or the administrator; Semi-anonymous: The participant has used a username that contains only their first name, thus partly identifiable by the other members of the platform and the administrator, or the participant has shared their first name and last name with the administrator only; Not anonymous: the participant has used a username that contains both their first name and last name, thus identifiable by both other members and the administrator. Many of the healthcare professionals did embrace the possibility of using a username which either contained their first name only or a representative username probably to promote more immediacy through this tool.

These preferences to anonymity were technically reinforced to preserve the right to anonymity yet facilitate networking and interaction between patients and families, and healthcare professionals.

## Discussion

To the best of our knowledge, there is no published research with a similar scope and type of results to allow accurate comparisons. Even though there are multiple social networks using Web 2.0 to source CI for clinical and patient support purposes [3] [32], they are either non-specifically focused on rare diseases or address patients/families (carers) or clinicians only. Within rare diseases, the closest projects in terms of mindset and methodology are the Share4Rare [23] [33] and the Patients Like Me projects [34].

The RARE-e-CONNECT project extends the application of Web 2.0 tools to both healthcare professionals (clinicians, laboratory scientists, allied healthcare professionals) and patients/families with rare disease diagnoses, separately and together. It provides technology-mediated opportunities for specialized information exchange and interaction to source multiple perspectives and create CI. Research-wise, it seeks to examine the uptake and integration of this social platform in the specific context of Cyprus, as a small EU country, against surrounding sociocultural, technological and policy factors to document ways of expanded, sustainable impact.

Prior to any technological design, we conducted a small-scale needs assessment survey with the “extreme users” of the network technology, confirming that Web 2.0 tools had significantly penetrated their work and life settings. Very importantly, the survey revealed aspects to consider e.g., possibilities for multidisciplinary collaboration, data sharing, requirements for a specialist repository and relevant levels of technological difficulty. Overall, this survey gave good insights into how the RARE-e-CONNECT proposal could align with the needs and current practices of our audiences (patients/families with rare disease diagnoses and healthcare professionals).

The main challenges identified during our meetings with healthcare professionals, patients/families, and national authorities, i.e., MOH and CyMA, were identified as the following: (1) absence of a reliable repository of reference centres or specialists^5^ by disease/disease group that they could immediately consult; (2) the absence of solid networking infrastructures that would allow for effective multidisciplinary collaboration, also between the private and the public sector; and (3) e-learning tools^6^ for CME. These needs aligned with our technological proposal and were materialized accordingly. At the remark of the National Representative at the ERN Board of Member States, a doctor-patient forum was created to avoid fragmentation and a “two camp” approach by the network technology-to-be. It was made clear that this forum would only be used to share disease-related information and not to discuss specific patient cases.

Following its launch, both clinicians and patients/families in our research stated that the RARE-e-CONNECT network technology was useful and relevant to their work and life needs.

Data showed that, in the case of healthcare professionals, RARE-e-CONNECT was likely to be most useful at the pre-diagnosis stage, where primary care physicians and specialists need to be aware of the possibility that a rare disease might be causing the symptoms observed and be informed about the specifics related to the disease [9] to direct the patient to the right specialist or service, i.e., genetic tests for a definitive diagnosis. This latter is the requirement in the vast majority of cases.

In the case of patients/families, data showed that RARE-e-CONNECT can equally serve patient education post-diagnosis as well as informed decision-making in emergency or bottleneck situations, where there is an increased need for disease-related information by an expert, patient experiential knowledge or psychosocial support.

However, both groups deemed that the integration/penetration of the RARE-e-CONNECT platform into professional and personal life was challenging. In many cases, it became clear that it was not the technology per se or the clinicians’ willingness to use it, but its ability to align with the demands of the workplace to enable committed participation for increased gains in job and life performance. The absence of infrastructure and policies around innovative activities to support clinical practice and patient care, such as telementoring/teleconsulting/shared care, was one important condition for participation [2].

Taken together, these findings suggest that, if organised within a socioculturally relevant, comprehensive framework, CI systems such as RARE-e-CONNECT could help establish a “second generation medicine” [35]. This latter would be characterized by: (1) patient to patient support to help cope with the emotional burden caused by the disease and adapt to the necessary life changes long-term [8], as well as (2) doctor-patient support where formal aspects of the disease will be explained to promote informed decision-making, including taboo aspects that are difficult to discuss in person [25]. Moreover, the results presented in this article, and other like-minded projects, suggest that CI platforms can greatly assist research on rare diseases, maximizing the potential in harnessing patient knowledge capital [33] [23] [22]. Indeed, patient experiential knowledge with the disease could yield unique insights of use to clinical practice [25].

Our findings seem to relate to many telehealth innovations, while some may even constitute new important insights for the implementation of a policy to define CI networks in rare diseases, subsequent action, and plans for sustainability [15] [17].

To improve the acceptance and output of such telehealth/telementoring tools, it is essential to:

1. provide adequate training/transition stages and technical assistance for using telementoring systems in line with the observed needs. Community managers are key to animating participation to avoid user drop out [12] [33].
2. introduce effective automatizations and artificial intelligence mechanisms to assist usage and data collection in order to fully harness the potential of BIG MIND technologies.
3. advocate for meaningful policy-making on the national and European levels to support the use of CI technologies, safely disseminating specialized information from the ERNs and other accredited international centres via national coordinators and otherwise.
4. construct strong organizational structures around these technologies to increase the information flow and the reliability of content through expert reviews, and thus counter harms and limitations inherent to telehealth applications [14].

## The strengths and limitations of this study

### Strengths

The strengths of this study include its comprehensive approach to building a collaborative information network for both rare disease patients/families and healthcare professionals. Participation reflected an unmet need and authentic interest by both known and unknown patients/families who engaged in this digital medium to meet other patients/families, as well as healthcare professionals who joined to find colleagues and know more about rare diseases.

At the time of this publication, the RARE-e-CONNECT platform was able to provide networking opportunities between Cyprus, Greece, the UK, Germany, Italy and Belgium for 78 different rare diseases, as well as for 17 clinical specialties, 3 laboratory specialties, 7 allied healthcare specialties. There are more than 7000 rare diseases currently identified at the global level whereas there is no national registry to provide a complete account of the rare diseases diagnosed on the island.

To the best of our knowledge, this is the first field work research analyzing the objective parameters that matter for the operation and long-term effectiveness of collaborative networks from the perspectives of both patients/families and healthcare professionals, as they derive from their real life experiences.

At the same time, this study has produced solid research data to showcase how experiential knowledge shared through patient stories can be used not only to produce disease-related information for patient psychosocial support and CME, but also to reinforce patient organisations’ advocacy work for policy-making and supportive infrastructure. Last but not least, it provided the opportunity for healthcare professionals and patient organisations to work and plan together for progress.

### Limitations

The participants in this research were patients/families and healthcare professionals with digital knowledge and literacy, access and previous experience with Internet and digital tools, citing the digital divide and creating a certain degree of bias;  thus a limitation of this study. This bias was also evident in the percentage of the country’s patient associations who participated in this project. All of the existing organized patient groups whose members were diagnosed with a rare disease were notified, as well as patient organizations with a mixed rare and non-rare disease membership. Nevertheless, only those associations with digitally-literate working staff registered and used the platform.

What is more, the various restrictions related to the pandemic e.g., accessibility and time, the implementation of the National Health System in Cyprus in 2019 and 2020, created multiple other priorities for both healthcare professionals and patients, families and patient representatives than to sufficiently use the RARE-e-CONNECT collaborative features to measure outcomes over time.

Therefore, this article describes the first phase of work-in-progress focused on developing a critical mass of patients/ families and healthcare providers for an effective knowledge-sharing network, promoting rare disease prevention and effective management according to the available curative options.

Further research is needed to evaluate the system at a larger scale with consistent participation from both Cyprus and abroad to provide a robust proof of concept of the ways it can serve deep knowledge construction on rare diseases, improving patient care in the long run.

## Conclusions

In this article, we researched the potential impact of the RARE-e-CONNECT network technology to achieve a positive impact on rare disease patient care in Cyprus.

As a whole, our findings represent many well-known challenges related to rare diseases worldwide, and showcase how such challenges may affect the uptake of telehealth tools and their potential to share specialised information across geographies. To mention but a few, (1) the limited rare disease expertise and awareness within the healthcare community, especially in small EU countries, (2) the absence of infrastructure e.g., nationally accredited centres of reference to (co-)produce specialized information for CME, share and offer telementoring, (3) the absence of policies to regulate telehealth and shared care, (4) the absence of a solid plan to coordinate efforts within the EU to develop the necessary synergies for the circulation of expert know-how into local communities.

Our findings suggest that, if organised into a comprehensive, coordinated and policy-regulated plan, CI networks, such as the one proposed in this article, can effectively support the circulation of expert know-how by creating international intersections to provide new rare disease know-how into local communities. Such intersections would help tackle public health problems related to timely diagnosis and effective management while saving resources through teleconsultation. Other studies on CI systems make similar arguments.

Certainly, the establishment of the ERNs constitutes a giant leap in identifying rare disease expertise and providing accreditation. This is an important step in addressing the shortage of expertise and advancing telehealth to alleviate patient care challenges that are due to geographical distances. CI platforms have a place in this effort. Many of the existing platforms represent a country or region. Thus, cross references (links) need to be established between CI systems across regions and languages, from a central directory for example. Cross-referencing could provide a more comprehensive networking and information service to both patients/families and healthcare professionals, maximizing the circulation of important and reliable information while promoting meaningful, needs-based interaction for substantial support and progress.

In this effort, special attention should be given to the evaluation of each system and the establishment of minimum requirements e.g., be available in English apart from the local language. Collaboration with local authorities, experts and patient organizations needs to be established for setting up the underlying organizational structure for the curation of content and its dissemination, based on the real needs of the local or regional audience. The EU Joint Action for the Integration of the ERNs into the national healthcare systems seems to be an ideal framework to encompass such an endeavour, reinforcing the efforts of the ERNs to disseminate the expert knowledge they produce, promote provider collaboration, patient participation, research and expansion.

## Data Availability

The authors confirm that the data supporting the findings of this study are available within the article [and/or] its supplementary materials. The full sets of anonymised quotes are available from the corresponding author on reasonable request.

## Footnotes

1 As per the definition provided by the World Health Organisation (2010), telehealth or eHealth is “the delivery of health care services, where distance is a critical factor, by all health care professionals using information and communication technologies for the exchange of valid information for diagnosis, treatment and prevention of disease and injuries, research and evaluation, and for the continuing education of health care providers, all in the interests of advancing the health of individuals and their communities”. For the purposes of this article, telehealth is differentiated from telemedicine, which refers to the provision of remote clinical services from a healthcare provider to a patient via telecommunication technologies. Although telehealth can also be taken to include the provision of clinical services via technology, in this article we understand that they are essentially offered in the presence of the patient’s treating physician or to the treating physician directly, taking the form of telementoring (see “Telemedicine in collaboration” in our data).

2 Endo-ERN, ERN-RND, ERN-NMD, ERN GENTURIS, ERN-ITHACA, ERN-Lung, EuroBloodNet.

3 Laboratory physicians and allied healthcare professionals also asked to be part of a rare disease network, initially designed for clinicians only. At this request, we developed a “more comprehensive registration profile” and they were included in the evaluation process as well.

4 Available at https://health.ec.europa.eu/publications/2022-eu4health-work-programme_en.

5 The specialist centre repository was created to include ERN members and other internationally-recognised centres of expertise, while a memorandum of collaboration was signed between the Ministry of Health and the Cyprus Alliance for Rare Disorders (Host Organisation of this project) for patient support in the context of crossborder healthcare, including information on the ERNs and other related activities. The specialist centre repository includes all ERN members from 2019 and is pending update to reflect new members and categorisations of expertise.

6 This was materialized as a webinar repository collecting key webinars taking place from CARD and other rare disease organizations, while also allowing HCPs to request webinar topics of interest to be organized locally, and automatic feeds of new publications in RDs, including clinical trials.

## List of abbreviations

CARD: Cyprus Alliance for Rare Disorders
CBPR/HCD: Community-Based Participatory Research and Human-Centred Design
CME: Continuing Medical Education
CyMA: Cyprus Medical Association
ERNs: European Reference Networks
CI: Collective Intelligence
MOH: Ministry of Health
UTAUT: Unified Theory of Acceptance and Use of Technology
